# Emilin-2 is a component of bone marrow extracellular matrix regulating mesenchymal stem cell differentiation and hematopoietic progenitors

**DOI:** 10.1186/s13287-021-02674-2

**Published:** 2022-01-10

**Authors:** Francesco Da Ros, Luca Persano, Dario Bizzotto, Mariagrazia Michieli, Paola Braghetta, Mario Mazzucato, Paolo Bonaldo

**Affiliations:** 1 SOSd Cell Stem Unit, Department of Translational Research, National Cancer Center CRO-IRCSS, 33081 Aviano, Italy; 2 Department of Molecular Medicine, University of Padova, Via Ugo Bassi 58/B, 35131 Padova, Italy; 3 Department of Women’s and Children’s Health, University of Padova, 35131 Padova, Italy; 4 IRP - Pediatric Research Institute, 35131 Padova, Italy; 5 SOSd Cell Therapy and High Dose Chemotherapy, National Cancer Center CRO- IRCCS, 33081 Aviano, Italy; 6 CRIBI Biotechnology Center, University of Padova, 35131 Padova, Italy

**Keywords:** Extracellular matrix, Bone marrow, Mesenchymal stem cells, Emilin-2

## Abstract

**Background:**

Dissection of mechanisms involved in the regulation of bone marrow microenvironment through cell–cell and cell–matrix contacts is essential for the detailed understanding of processes underlying bone marrow activities both under physiological conditions and in hematologic malignancies. Here we describe Emilin-2 as an abundant extracellular matrix component of bone marrow stroma.

**Methods:**

Immunodetection of Emilin-2 was performed in bone marrow sections of mice from 30 days to 6 months of age. Emilin-2 expression was monitored in vitro in primary and mesenchymal stem cell lines under undifferentiated and adipogenic conditions. Hematopoietic stem cells and progenitors in bone marrow of 3- to 10-month-old wild-type and Emilin-2 null mice were analyzed by flow cytometry.

**Results:**

Emilin-2 is deposited in bone marrow extracellular matrix in an age-dependent manner, forming a meshwork that extends from compact bone boundaries to the central trabecular regions. Emilin-2 is expressed and secreted by both primary and immortalized bone marrow mesenchymal stem cells, exerting an inhibitory action in adipogenic differentiation. In vivo Emilin-2 deficiency impairs the frequency of hematopoietic stem/progenitor cells in bone marrow during aging.

**Conclusion:**

Our data provide new insights in the contribution of bone marrow extracellular matrix microenvironment in the regulation of stem cell niches and hematopoietic progenitor differentiation.

**Supplementary Information:**

The online version contains supplementary material available at 10.1186/s13287-021-02674-2.

## Introduction

Bone marrow (BM) is a heterogeneous tissue involved in hematopoiesis. Although hematopoietic stem cells (HSC) exert the main role in repopulating immune cells and all blood lineages, their activity can be modulated by different factors related to BM microenvironment. Mesenchymal stem cells (MSC), osteoblasts, adipocytes, endothelial cells, cytokines and extracellular matrix (ECM) proteins provide a number of signals modulating HSC function in BM. Literature work highlighted the importance of BM-MSC [1] and the proper balance between osteogenic and adipogenic differentiation, which can influence in opposite ways HSC function and activity [2–5].

The importance of MSC in the maintenance of BM homeostasis is further highlighted by several pathological conditions in which these cells can be modulated by tumor cells, like multiple myeloma or leukemia cells, to mold a tumor-supporting microenvironment through secretion of exosomes and matrix metalloproteinases or modification of osteogenic/adipogenic potential [6–8]. Alterations of BM microenvironment occur not only in multiple myeloma and leukemia, but also in myelodysplastic syndromes (MDS), a heterogeneous group of disorders characterized by anomalous growth of HSC and their abnormal differentiation in hematopoietic progenitor cells (HPC) as common myeloid progenitors (CMP), granulocyte myeloid progenitors (GMP) and megakaryocyte–erythrocyte progenitors (MEP) [9]. In particular, studies aimed at the characterization of hematopoietic stem and progenitor cell populations in MDS patients correlating with the risk of acute myeloid leukemia (AML) revealed an increased number of HSC and CMP coupled with a decreased amount of GMP in MDS with low risk of AML, whereas an expansion of GMP was observed in patients with high risk of AML onset [10–12].

It has been established that the ECM compartment of BM includes proteoglycans, collagens, fibronectin and tenascin-C [13–19], and that these ECM components influence both normal hematopoiesis and the development of hematological malignancies, such as lymphoma, leukemia and multiple myeloma (reviewed in [20]. Interestingly, recent studies of the matrisome [21] of AML patients highlighted a number of ECM-related genes with potential involvement in tumor onset and progression [22, 23]. Among these, *EMILIN2,* a gene coding for an ECM glycoprotein of the Emilin/Multimerin family [24, 25], belonged to the group of most deregulated genes in AML patients when compared to healthy donors [23]. Notably, independent studies demonstrated that Emilin-2 plays a critical role in modulating tumor cell apoptosis and angiogenesis [26–28].

Starting from these literature evidence, we investigated the role of Emilin-2 in murine BM, taking advantage of the availability of an *Emilin2* null mouse model. Our results indicate that Emilin-2 is a key component of murine BMECM involved in MSC and hematopoietic differentiation in an age-dependent manner.

## Results

### Emilin-2 is an ECM component of adult mouse BM

**Fig. 1:**
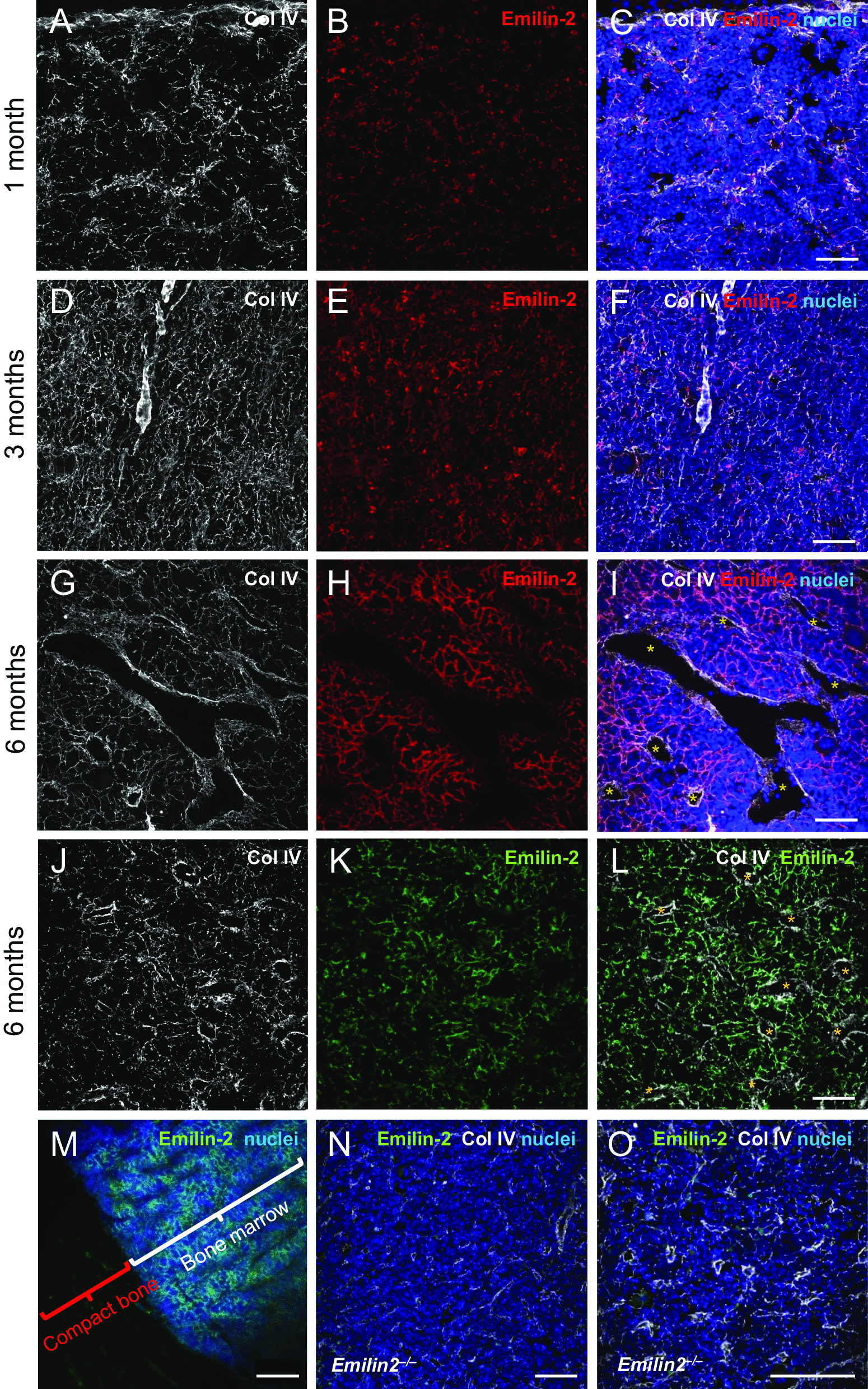
Emilin-2 distribution in BM of adult mice. **A**–**I** Representative immunofluorescence for Emilin-2 (red) and Collagen IV (gray) in BM of femur of 1-month-old (**A**–**C**), 3-month-old (**D**–**F**) and 6-month-old (**G**–**I**) wild-type mice. Asterisks in panel I indicate sinusoids and vessels. **J**–**L** Representative immunofluorescence for Emilin-2 (green) and Collagen IV (gray) in BM of tibia of 6-month-old wild-type mice. Asterisks in panel L indicate sinusoids and vessels. **M** Whole-mount immunofluorescence staining for Emilin-2 (green) in BM of femur of 6-month-old wild-type mice. BM and compact bone areas are indicated. **N**, **O** Representative immunofluorescence for Emilin-2 (green) and Collagen IV (gray) in BM of femur of 6-month-old *Emilin2* null mice. In panels **C**, **F**, **I**, **M**, **O**, nuclei were stained with Hoechst (blue). Scale bar, 100 μm. Col IV, Collagen IV. See also Additional file 1: Fig. S1

Immunofluorescence microscopy of frozen sections of decalcified femurs and tibiae of 1- to 6-month-old mice showed that Emilin-2 is deposited in the BM stroma (Fig. 1A–L). A strong Emilin-2 signal was displayed by the diaphyseal regions of both femur (Fig. 1H) and tibia (Fig. 1K), starting from the endosteal region to central vessel area (Additional file 1: Fig. S1A), but not in compact bone (Fig. 1M; Additional file 1: Fig. S1A). Emilin-2 deposition in BM-ECM increased during postnatal life, as shown by staining at 1, 3 and 6 months (Fig. 1B, E, H),forming a meshwork that partially co-localized with Collagen IV, a well characterized BMECM protein, and surrounding various sets of cells (Fig. 1C, F, I). As expected, Emilin-2 staining was absent in BM sections of *Emilin2* null mice (Fig. 1N, O; Additional file 1: Fig. S1E), thus confirming the specificity of the immunolabeling. Interestingly, Emilin-2 labeling in BM resembled Collagen IV deposition [16], but also showed a distinctive pattern, since Collagen IV staining surrounded arteriolar vessels and sinusoids (Fig. 1A, D, G, J), whereas Emilin-2 signal was absent in both structures(Fig. 1C, F, I, L). Higher magnifications confirmed that Emilin-2 deposition in sinusoids and vessels was found in the proximity of Collagen IV staining, but did not co-localize with it (Additional file 1: Fig. S1B–D).

### Emilin-2 is expressed by MSC and is down-regulated during adipogenic differentiation

**Fig. 2:**
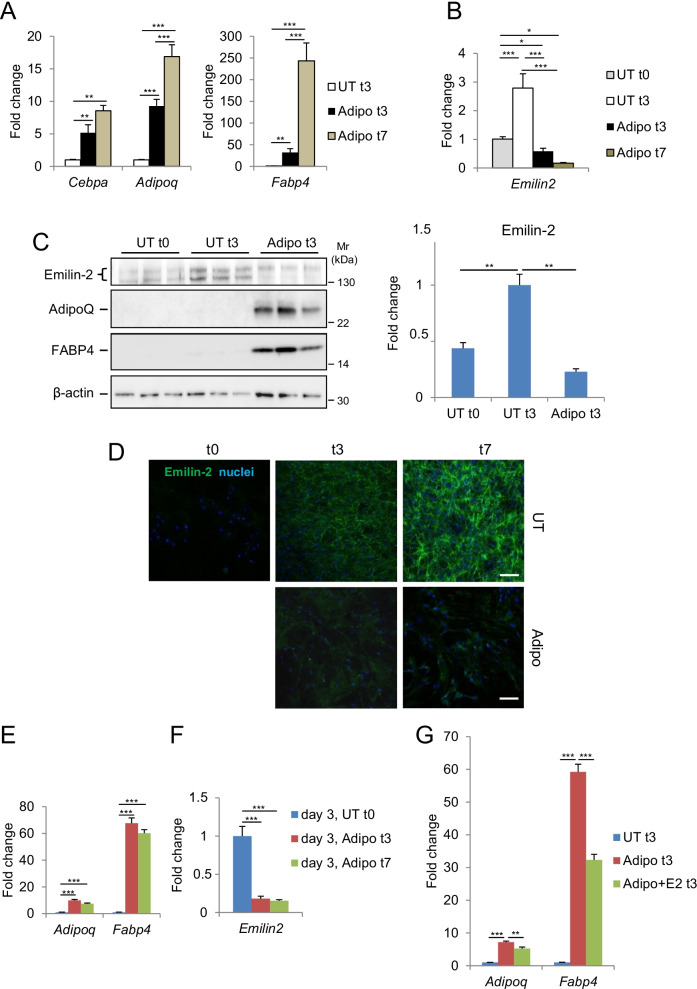
Emilin-2 expression in ST2 cells during in vitro adipogenic differentiation. **A** mRNA levels of adipogenic markers in ST2 cells under non-differentiating conditions (UT t3) or after 3–7 days of treatment with adipogenic stimuli (Adipo t3 and t7), as determined by RT-qPCR. mRNA levels are shown as fold change compared to UT t3 condition (*n* = 3–9; *P < 0.05;**P < 0.01; ***P < 0.001). **B** RT-qPCR analysis of mRNA levels of Emilin-2 in ST2 cells under non-differentiating conditions (UT t0 and t3) and after 3 and 7 days of treatment with adipogenic stimuli (Adipo t3 and t7). mRNA levels are shown as fold change compared to UT t0 condition (*n* = 3–9; *P < 0.05; ***P < 0.001). **C** Western blot analysis for Emilin-2, AdipoQ and FABP4 proteins in ST2 cells under non-differentiating condition (UT t0 and t3) and after 3 days of adipogenic stimuli (Adipo t3). Three independent samples are shown for each condition. β-actin was used as a protein loading control. The graph on the right show densitometric quantification for Emilin-2, as determined by two independent experiments. Protein levels are shown as fold change compared to UT t3 condition (*n* = 3–6; *P < 0.05;**P < 0.01) **D** Immunofluorescence for Emilin-2 (green) in ST2 cells at 0, 3 and 7 days culture in untreated conditions (UT) or in the presence of adipogenic stimuli (Adipo). Nuclei were stained with Hoechst (blue). Scale bar, 100 µm. **E**, **F** RT-qPCR analysis of the mRNA levels for AdipoQ, FABP4 (**E**) and Emilin-2 (**F**) in ST2 cells cultured for 3 days before adipogenic treatment (day 3, UT t0) and then treated for 3 and 7 days with adipogenic stimuli (day 3, Adipo t3 and t7). mRNA levels are shown as fold change compared to day 3, UT t0 condition(*n* = 3; ***P < 0.001). **G** Relative mRNA levels of AdipoQ and FABP4 in ST2 cells cultured under non-differentiating conditions (UT t3) or after 3 days of adipogenic stimulus in cells pre-treated (Adipo + E2 t3) or not (Adipo t3) with purified Emilin-2 protein. mRNA levels are shown as fold change compared to UT t3 condition (*n* = 3; **P < 0.01; ***P < 0.001). See also Additional file 1: Figs. S1 and S2

Within BM microenvironment, MSC are major actors in regulating BM homeostasis [4]. In particular, the unbalance toward adipogenic differentiation of BM-MSC, which can occur during aging or pathological conditions, negatively impacts HSC quiescence, proliferation and differentiation [2, 3, 5]. The abundant deposition of Emilin-2 in BM ECM prompted us to investigate its expression in stromal BM cells, and in particular in MSC under undifferentiated and differentiated conditions. Using the ST2 cell line as a surrogate of murine BM-MSC [29], we evaluated Emilin-2 expression at the RNA and protein levels both in non-differentiating conditions and following adipogenic differentiation (Fig. 2A–D). ST2 cells maintained under non-differentiating conditions displayed a progressive increase of Emilin-2 mRNA and protein levels during culture (Fig. 2B–D). To assess whether Emilin-2 expression is modulated during MSC differentiation, we administered adipogenic stimuli for 3 and 7 days. Emilin-2 mRNA and protein levels rapidly decreased during the first days of differentiation (Fig. 2B–D), in parallel with the concurrent increase of adipogenic markers (Fig. 2A, C). Indeed, while the adipogenic markers AdipoQ, C/EBP-alpha, and FABP4 were increasing at both 3 and 7 days of differentiation, Emilin-2 displayed a marked decrease at day 3, and even more at day 7 of differentiation (Fig. 2B, C). Immunostaining of ST2 cultures confirmed that Emilin-2 deposition increased and became progressively more abundant during non-differentiating conditions, but was very low following adipogenic differentiation (Fig. 2D).

Although this set of data indicated that Emilin-2 expression is reduced during adipogenic differentiation, in the above experiments we started treating cells one day after plating, when Emilin-2 deposition is still low (Fig. 2D, t0 condition; Additional file 1: Fig. S2A). To understand whether Emilin-2 is down-regulated during adipogenesis, we induced adipogenic differentiation after 3 days from plating (Additional file 1: Fig. S2B), a time point in which Emilin-2 expression is much higher in undifferentiated ST2 cells (Additional file 1: Fig. S2C). Again, adipogenic differentiation was confirmed by the increased expression of AdipoQ and FABP4 following administration of adipogenic stimuli (Fig. 2E). Notably, Emilin-2 mRNA levels were high in undifferentiating conditions but decreased of about 80% after 3 and 7 days of adipogenic differentiation (Fig. 2F), supporting the concept that *Emilin*2 expression is down-regulated during adipogenic differentiation. To assess whether *Emilin*2 was down-regulated due to a potentially inhibitory effect it may exert on adipogenesis, we cultured ST2 cells in the presence of purified Emilin-2, added in the culture medium one day before starting the experiment (Additional file 1: Fig. S2D). Of note, pre-treatment of cells with Emilin-2 led to significantly decreased levels of AdipoQ and FABP4 transcripts after 3 days from adipogenic differentiation, when compared to cells maintained in the same conditions but without Emilin-2 (Fig. 2G),thus supporting an inhibitory effect for Emilin-2 on the adipogenic differentiation of BM-MSC.

### Primary BM-MSC regulate Emilin2 expression and deposition during adipogenesis

**Fig. 3:**
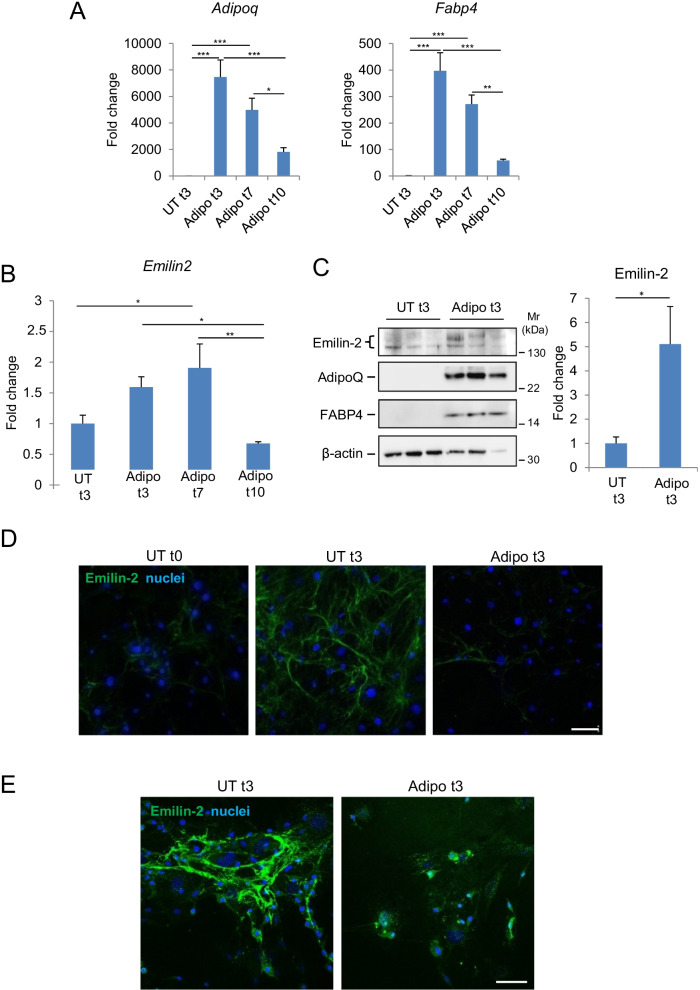
Emilin-2 expression during in vitro adipogenic differentiation of primary BM-MSC. **A**, **B** RT-qPCR analysis of the mRNA levels for the adipogenic markers AdipoQ (**A**, left) and FABP4 (**A**, right) or for Emilin-2 (**B**) in primary murine BM-MSC under non-differentiating conditions (UT t3) or treated for 3, 7 and 10 days with adipogenic stimuli (Adipo t3, t7 and t10). mRNA levels are shown as fold change compared to UT t3 condition (*n* = 6; *P < 0.05;**P < 0.01; ***P < 0.001). **C** Western blot analysis for Emilin-2, AdipoQ and FABP4 proteins in primary murine BM-MSC under non-differentiating conditions (UT t3) and after 3 days of adipogenic differentiation (Adipo t3). Three independent samples are shown for each condition. β-actin was used as a protein loading control. The graph on the right show the densitometric quantification for Emilin-2, as determined by two independent experiments. Protein levels are shown as fold change compared to UT t3 condition (*n* = 4; *P < 0.05). **D** Representative immunofluorescence for Emilin-2 (green) in primary murine BM-MSC cultures in control conditions (UT t0) and after 3 days in non-differentiating (UT t3) or adipogenic differentiating (Adipo t3) conditions. Scale bar, 100 μm. Nuclei were stained with Hoechst (blue). **E** Immunofluorescence for Emilin-2 (green) in primary murine BM-MSC cultures maintained for 3 days in non-differentiating (UT t3) or adipogenic differentiating (Adipo t3) conditions, and subjected to permeabilization with Triton X-100 before immunostaining. In non-differentiating conditions, most Emilin-2 labeling is found in the ECM in the form of an organized fibrillar network, whereas in cultures subjected to adipogenic differentiation Emilin-2 reactivity is found inside the cells in the form of dots scattered throughout the cytosol. Nuclei were stained with Hoechst (blue). Scale bar, 100 μm. See also Additional file 1: Figs. S1, S2 and S3

To further understand the modulation of Emilin-2 expression during MSC differentiation, we studied primary BM-MSC isolated from wild-type mice. After three passages to reduce the presence of contaminating leukocytes, we first confirmed the capability of these cells to differentiate in the presence of adipogenic stimuli for different times (Additional file 1: Fig. S2E). Culture of primary BM-MSC in adipogenic differentiation medium for 3 to 10 days led to a marked increase of AdipoQ and FABP4 at both mRNA and protein levels, when compared to cells maintained in non-differentiating conditions (Fig. 3A, C). As with ST2 cultures, Emilin-2 was expressed by primary undifferentiated BM-MSC and its deposition became more abundant during culture (Fig. 3B–D). At difference from ST2 cells, Emilin-2 mRNA levels increased during the first days of adipogenic differentiation, whereas a trend toward down-regulation of Emilin-2 mRNA levels was observed only after 10 days of adipogenic differentiation (Fig. 3B). However, immunodetection of Emilin-2 protein in BM-MSC at day 3 of culture in adipogenic differentiation medium revealed a weaker extracellular staining when compared to cultures maintained in standard medium (Fig. 3D). Cell permeabilization before immunostaining showed the presence of intracellular Emilin-2 positive dots in differentiating BM-MSC cultures (Fig. 3E), and immunodetection at longer times of differentiation confirmed the drop in Emilin-2 levels (Additional file 1: Fig. S3). These data indicate that Emilin-2 deposition is down-regulated in primary BM-MSC during adipogenic differentiation but, at difference from immortalized ST2 cells, the first step in modulation of Emilin-2 levels is a decrease in protein secretion, followed by a progressive drop in gene expression and protein deposition.

### In vivo ablation of Emilin-2 impairs HSC pool and affects the differentiation of hematopoietic precursors during aging

**Fig. 4:**
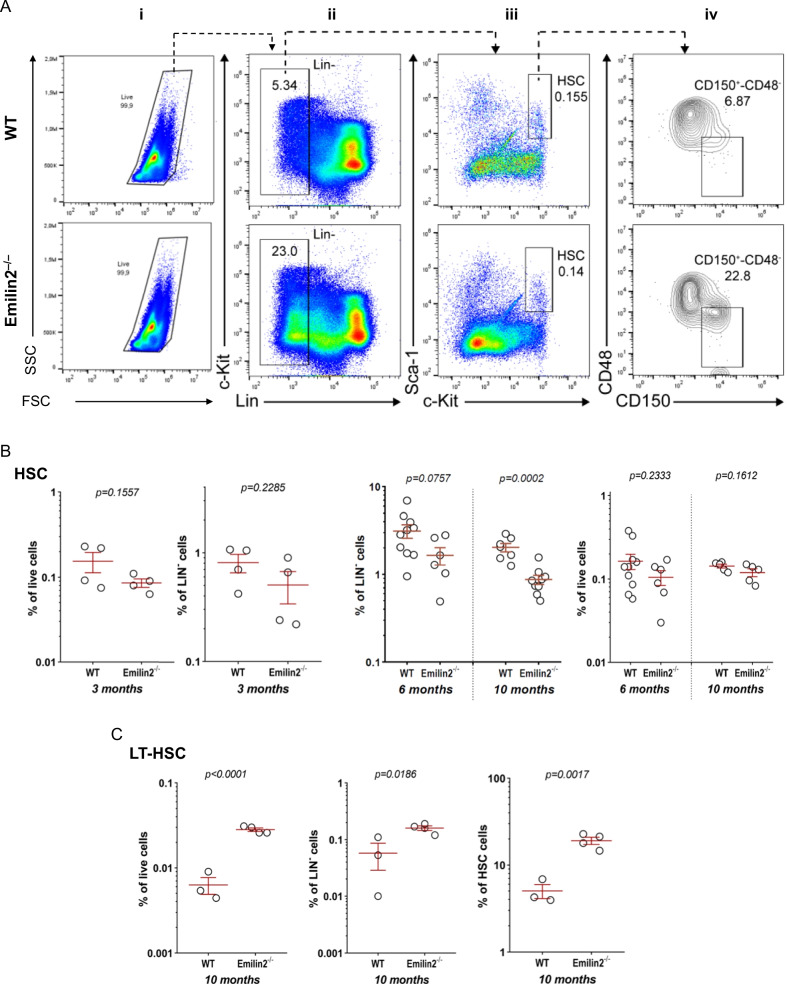
Frequency of in vivo HSC and LT-HSC hematopoietic progenitors in adult wild-type and Emilin-2 null mice. BM were isolated from wild-type and *Emilin2*^*−*/*−*^ mice of 3,6 and 10 months of age and subjected to flow cytometry with different antibodies. **A** Representative flow cytometry analysis of HSC and LT-HSC in BM of 10-month-old mice, based on the identification of lineage-negative (Lin^*−*^) cells and of Sca1, c-Kit, CD150 and CD48 markers. Live cells, identified with side scatter (SSC; granularity) and forward scatter (FSC; cell size) parameters (plot i), negative for Lin^*−*^marker (plot ii), were selected and analyzed for Sca1 and c-Kit signal (plot iii). Double positive Sca1^+^/c-Kit^+^ events were identified as HSC (squared areas of plot iii) and subjected to further analysis based on CD150 and CD48 signals to identify LT-HSC (Cd150^+^/CD48^*−*^; squared area of plot iv). **B** Percentages of HSC, calculated on total live cells and on Lin^*−*^ cells, n BM of 3-month-old (wild type, *n* = 4;*Emilin2*^*−*/*−*^, *n* = 4), 6-month-old (wild-type, *n* = 10; *Emilin2*^*−*/*−*^, *n* = 6) and 10-month-old (wild-type, *n* = 7; *Emilin2*^*−*/*−*^, *n* = 9) mice. **C** Percentage of LT-HSC, calculated on either total live cells, Lin^*−*^ cells and HSC cells, in BM of 10-month-old (wild-type, *n* = 3; *Emilin2*^*−*/*−*^, *n* = 4) mice

**Fig. 5:**
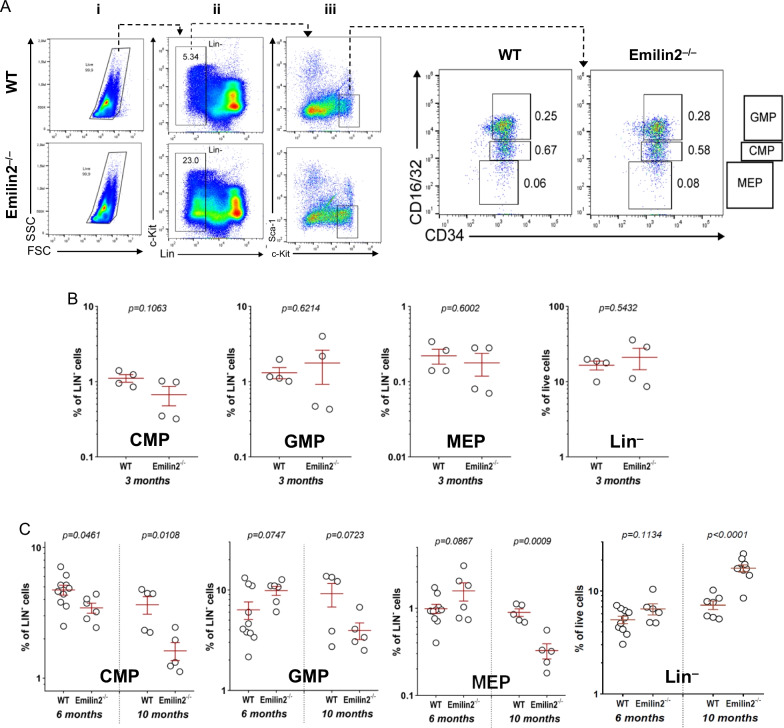
Frequency of in vivo hematopoietic progenitors in adult wild-type and Emilin-2 null mice. BM were isolated from wild-type and *Emilin2*^*−*/*−*^ mice of 3, 6 and 10 months of age and subjected to flow cytometry with different antibodies. **A** Representative flow cytometry analysis of hematopoietic progenitors. Sca1^*−*^/c-Kit^+^ events (plot iii of Fig. 4A) were subjected to further analysis, based on CD16/32 and CD34 signals, to identify GMP (CD16/32^+^/CD34^+^; top square), CMP (CD16/32^*−*^/CD34^+^; middle square) and MEP (CD16/32^*−*^/CD34^*−*^; bottom square). **B** Percentages of CMP, GMP and MEP in Lin^*−*^ subpopulation and percentages of Lin^*−*^ cells on total live cells in BM of 3-month-old (wild type, *n* = 4; *Emilin2*^*−*/*−*^, *n* = 4). **C** Percentages of CMP, GMP and MEP in Lin^*−*^ subpopulation and percentages of Lin^*−*^ cells on total live cells in BM of 6-month-old (wild-type, *n* = 10; *Emilin2*^*−*/*−*^, *n* = 6) and 10-month-old (wild type, *n* = 5–7; *Emilin2*^*−*/*−*^, *n* = 5–9) mice. WT, wild-type. See also Additional file 1: Fig. S4

Several literature data have linked abnormal HSC differentiation and altered distribution of hematopoietic progenitors in BM with alterations of ECM microenvironment (reviewed in [30]. Based on the abundant deposition of Emilin-2 in BM ECM, and taking advantage of the availability of an Emilin-2 null (*Emilin2*^*−*/*−*^) mouse model [28], we investigated whether the absence of this protein may affect the frequency of hematopoietic stem cells (HSC) and of hematopoietic progenitor cells (HPC)—including common myeloid progenitors (CMP), granulocyte myeloid progenitors (GMP) and megakaryocyte–erythrocyte progenitors (MEP)—during adulthood. Using a cytometric approach based on specific markers for the various classes of hematopoietic progenitors (Additional file 1: Fig. S4), we detected a progressive change in HSC and progenitor populations from 3 to 10 months of age in *Emilin2*^*−*/*−*^ mice, when compared to age-matched wild-type mice (Figs. 4A–C, 5A–C). At 3 months of age, no differences were observed in the frequency of cell populations (Figs. 4B, 5B), while at 6 months *Emilin2*^*−*/*−*^ BM displayed a trend toward decreased HSC pool versus lineage-negative (Lin-) population, but not versus total live cells(Fig. 4B). At 10 months of age, *Emilin2*^*−*/*−*^ mice showed a dramatic decrease of HSC, CMP and MEP frequencies and a trend toward a reduction of GMP abundance in Lin- population(Figs. 4B, 5C),pointing at an impairment in the maintenance of the proper pool of hematopoietic progenitors and an unbalance in the differentiation process.

**Fig. 6:**
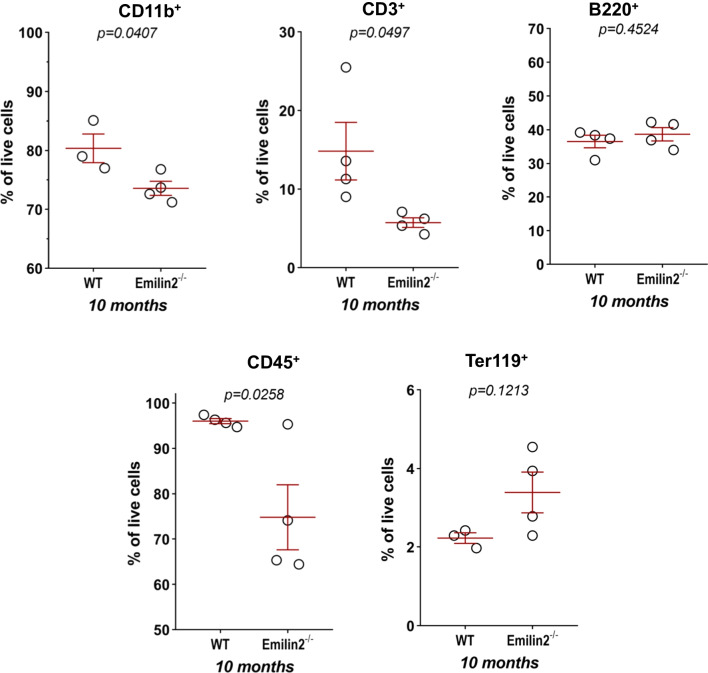
Frequency of in vivo differentiated blood cells in BM of adult wild-type and Emilin-2 null mice. Percentages, calculated on total live cells, of CD11b^+^, CD3^+^, B220^+^, Ter119^+^ and CD45^+^ cells in BM isolated from wild-type (n = 4) and *Emilin2*^*−*/−^(n = 4) mice of 10 months of age. WT, wild-type

Further characterization of the HSC subpopulation revealed that 10-month-old *Emilin2*^*−*/*−*^ mice have higher frequencies of long term-(LT)-HSC among live cells, total HSC and Lin-cells (Fig. 4C). Moreover, the decrease of the HSC pool in Lin- subpopulation, but not in total live cells, was associated with a progressive increased frequency of Lin- cells in *Emilin2*^*−*/*−*^ BM from 3 to 10 months of age (Fig. 5B, C). These data, combined with a significant decreased frequency of CD45+ cells in 10-month-old *Emilin2*^*−*/*−*^BM (Fig. 6), supported the concept of progressive impairment of differentiation in the hematopoiesis process. In agreement with this, further analyses with lymphoid and myeloid differentiation markers revealed significantly decreased amounts of CD11b+ (monocyte/macrophage) and CD3+ (T cell) pools, but not of Ter119+ (erythroid) and B220+ (B cell) pools, in BM of 10-month-old *Emilin2*^*−*/*−*^ mice (Fig. 6).

**Fig. 7:**
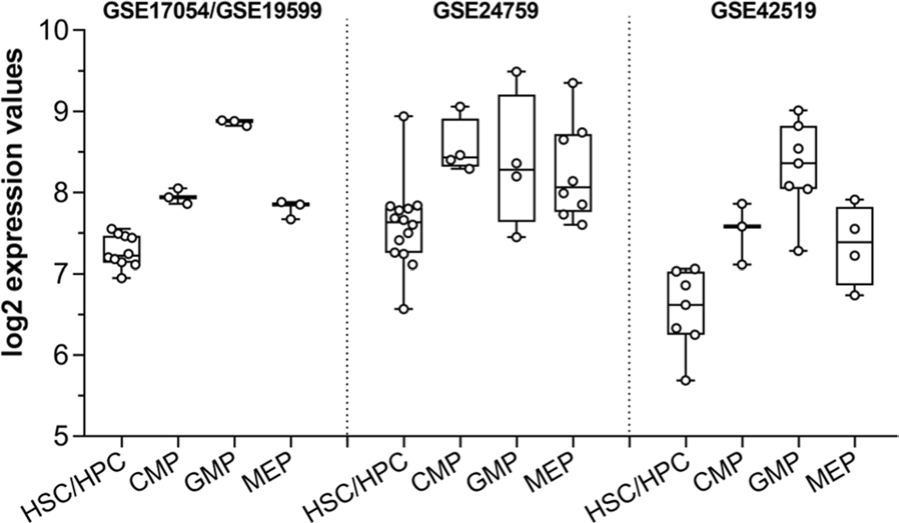
Emilin-2 expression in human hematopoietic cells from publicly available datasets. Boxplot displaying the log_2_ transformed expression values for Emilin-2 in HSC, HPC and myeloid progenitors (CMP, GMP, MEP), as retrieved from different human hematopoietic (GSE17054, GSE19599, GSE24759, GSE42519) datasets. Normalized expression data were retrieved from BloodSpot database [54], without the application of any additional parameter

These findings suggested a possible link between the altered frequencies of HSC and progenitors in Emilin-2 null BM and Emilin-2 expression in the respective lineages. Interestingly, analysis of three human gene expression datasets in the hematopoietic lineage (GSE17054/GSE19599, GSE24759 and GSE42519) revealed that CMP and GMP, two of the HPC populations with decreased frequencies in 10-month-old *Emilin2*^*−/−*^BM, express higher level of Emilin-2 when compared to HSC and MEP (Fig. 7).

## Discussion

The BM microenvironment is essential for regulating hematopoiesis and involves several players belonging to both cell and ECM compartments. The cell compartment includes a wide heterogeneity of hematopoietic and stromal cells, such as HSC, HPC, MSC, osteolineage cells, adipocytes, endothelial/sinusoidal cells and immune cells, whose roles in regulating BM homeostasis and HSC niche has been well understood in recent years (for reviews, see [31–33]. Conversely, knowledge about the ECM compartment is still scant and largely based on older studies carried out in the last century [13–15, 18, 19], with only few most recent findings [16, 17, 34]. In particular, although the impact of ECM in influencing BM functions is increasingly recognized, there is still scarce information about the role of specific ECM components in the regulation of BM homeostasis.

In this study, we show that Emilin-2, an ECM protein belonging to the Emilin/Multimerin family [24, 25], is a distinctive component of murine BM microenvironment and exerts a role in modulating hematopoiesis and MSC differentiation. Our data show that Emilin-2 is present in the ECM of both femur and tibia with a distinctive pattern, which extends from the endosteal district to the central region of BM. Interestingly, the Emilin-2 matrix network surrounds group of cells of different types and shapes, recapitulating in part Collagen IV matrix deposition [16], but with the exclusion of vessels and sinusoids.

To explore the role of Emilin-2 in BM, we characterized its expression and secretion by MSC, considering the role of MSC in modulating BM homeostasis through the balance between osteogenic and adipogenic differentiation [2, 3, 5]. Our data show that not only Emilin-2 is expressed and secreted by both ST2 MSC cell line and primary BM-derived MSC, but it also exerts a remarkable effect in modulating adipogenic differentiation. Indeed, we found that Emilin-2, besides being down-regulated and decreased in the ECM of MSC undergoing adipogenic differentiation, also exhibits an inhibitory activity when supplied as a purified protein to MSC subjected to adipogenic stimuli, leading to markedly reduced levels of the AdipoQ and FABP4 markers. These findings are of pivotal relevance, as they unveil a role for Emilin-2 in the modulation and balance of osteogenic vs adipogenic differentiation of MSC, thus expanding the landscape of molecular players, different from cytokines and growth factors [35, 36], involved in their homeostasis and consequently in HSC modulation [4]. These data are also consistent with a number of literature studies showing that BM adipocytes are not simple tissue filler, but they act as critical regulators of HSC quiescence, proliferation and differentiation [2, 3, 5].

Of note, in vivo ablation of Emilin-2 also affects hematopoiesis, since our data show that *Emilin2* null mice display a progressive age-related drop of the frequency of hematopoietic progenitors, coupled with an expansion of the immature (Lin-) cell population and a decreased abundance of mature monocytes and lymphocytes when compared to wild-type mice. The defective capability of *Emilin2* null mice to maintain hematopoiesis during aging may be related to a higher increase of adipose tissue in BM of Emilin-2 deficient animals, in agreement with the unbalanced adipogenic differentiation that is observed during aging [2]. Such aspect is worthy to be investigated in further detail in the future, also monitoring the respective activity of constitutive vs regulated BM adipose tissue, which are known to differently affect hematopoiesis and whose composition is dynamically regulated in response to a variety of physiological and pathological conditions [37].

The detailed understanding of mechanisms regulating adipogenic differentiation has increasing relevance not only in the physiological control of BM homeostasis, but also in the etiopathology of hematological malignancies, such as multiple myeloma [6, 38–40], and in the recovery after tumor treatment with chemotherapy and radiotherapy cycles. Indeed, inhibition or induction of adipogenesis in BM was found to elicit positive and negative effects, respectively, in hematopoietic recovery after chemotherapy [5]. Radiotherapy treatment of different types of cancer, not directly related to bones, increases BM adipose tissue mass together with increased osteoclast activities, leading to worsening of bone parameters, such as trabecular bone volume, in both humans and rodents [41–44]. As a fibrillar component of ECM [45], it is conceivable that Emilin-2 may be involved in the regulation of BM biomechanical properties, which are well known modulators of MSC and HSC fate decision [46–48].

## Conclusions

The present data throw light on the modulation of hematopoiesis by a distinctive ECM component of BM microenvironment, opening new perspectives about the role of this ECM protein in the onset and progression of BM disorders characterized by altered HPC frequency [9]. Indeed, the BM of *Emilin2* null mice displays a decreased amount of CMP, GMP and MEP, and an expansion of immature cell populations. Literature work showed that deregulation of PU.1, a transcription factor mainly expressed by CMP and GMP, is a key molecular event in the initiation of AML [49]. Interestingly, in a comprehensive ChIP-Seq-based study for PU.1 target genes, *Emilin2* was identified as a target gene of PU.1 [50], thus suggesting a possible mechanistic relationship between Emilin-2 and occurrence of hematopoietic alterations in the BM. In addition, studies aimed at the characterization of the transcription profiles of ECM-related genes in AML patients identified *Emilin2* as part of the signature of leukemia precursor cells and AML [23], thus pointing at an involvement of Emilin-2 in AML development. Of note, based on the analysis of RNA expression data in leukemia precursor cells, in AML cells and in the BM of AML patients, *Emilin2* belongs to the group of most deregulated genes in AML patients [22, 23].

Altogether, our data show that Emilin-2, an ECM protein known to be involved in angiogenesis and tumor modulation [26–28], is a component of BM microenvironment involved in multiple functions in BM homeostasis, spanning from regulation of MSC differentiation to modulation of HSC pool and hematopoietic progenitor cell frequency. These findings pave the way for future studies aimed at dissecting in detail the roles of this ECM component in hematopoiesis sand its potential contribution in regulating adipocyte accumulation in BM during aging, as well as in the pathogenesis of MDS and AML. Such studies will provide valuable information for the detailed understanding of the roles of BM ECM in physiological and pathological conditions, and as well as for elucidating the contribution of distinct ECM players in the control of microenvironment and stem niches in BM tissue.

## Material and methods

### Mice

Wild-type and *Emilin2* null mice in the C57/BL6N background [28] were bred in Specific Pathogen Free animal facility. The manipulation of animals and the harvest of organs were approved by an internal ethics committee and by the Italian Health Ministry (reference code D2784.N.XJY).

### Immunofluorescence

Bones of 1-,3- and 6-month-old mice were fixed in 4% paraformaldehyde at 4 °C overnight, washed in phosphate-buffered saline (PBS) and embedded in OCT after decalcification for two weeks with a 14% EDTA solution. Ten µm slices were obtained with cryostat and processed for immunofluorescence. Slides of ST2 cells and primary MSC were fixed in 4% paraformaldehyde at room temperature for 5 min, washed in PBS and maintained at 4 °C until use. In brief, tissue slices were permeabilized using cold methanol/acetone for 5 min, washed three times in PBS, and incubated for 1 h with 4% bovine serum albumin (BSA) in PBS, to block unspecific binding of the antibodies. Samples were incubated overnight at 4 °C with the following primary antibodies diluted with 1% BSA in PBS: guinea pig anti-Emilin-2 (1:200 [45], rabbit anti-Emilin-2 (1:200 [45], rabbit anti-Collagen IV (1:200, Millipore). Slices were washed three times in PBS and then incubated for 1 h with the following secondary antibodies: anti-rabbit Cy3, anti-rabbit Cy5.5, anti-guinea pig Cy3 and anti-guinea pig 488 (all 1:400; Jackson ImmunoResearch). Hoechst 33258 (Invitrogen) was used for nuclear staining. After three washes in PBS, slices were mounted in glycerol 80%.

### Cell culture and treatments

The ST2 cell line was used as MSC surrogate [29] at passages from p3 to p5 and cultured in DMEM (Gibco), 10% fetal bovine serum (FBS), 1% glutamine, 1% penicillin/streptomycin. Primary BM-derived MSC were isolated from 4- to 8-week-old wild-type mice as described [51]. Primary cell cultures were maintained distinct for each experiment. Briefly, femur and tibia were cleaned from muscles and tendons and BM was flushed out with Alpha-MEM (Gibco) supplemented with 10% FBS, 1% penicillin/streptomycin, 1% glutamine and 10 ng/ml FGF2 [52]. After five days in culture, cells were split by incubation for 2 min with 0.25% trypsin (Invitrogen), to enrich the culture for MSC [51], and cultivated in the same medium. To induce adipogenic differentiation, ST2 cells and primary MSC were cultured for 10 days in Alpha-MEM supplemented with 10% FBS, 1% glutamine, 1% penicillin/streptomycin, 10^−6^ M dexamethasone (Sigma), 50 μM indomethacin (Sigma) and 0.5 mM 3-isobutyl-1-methylxanthine (Sigma), changing the medium every three days. Where indicated, ST2 cells were plated for one day in the presence of purified Emilin-2 [28], added as soluble protein in culture medium at a final concentration of 200 ng/ml. Oil Red O (Sigma) staining was performed after 18 days of adipogenic differentiation in BM-MSC as recommended by the manufacturer.

### Flow cytometry

Flow cytometry was performed using CytoFLEX flow cytometer (Beckman Coulter) and FlowJo software (Tree Star). BM from femur and tibia was flushed with 5 ml PBS using a 26/27G needle, and filtered through a 70-μm cell strainer. The cell strainer was washed with other 3 ml of PBS to increase yield, before centrifugation at 1,500 rpm for 5 min. Erythrocytes were removed via hypotonic shock, by resuspending the pellet with 3 ml of 0.2% NaCl for 30 s and then adding an equal volume of 1.6% NaCl. BM cells were centrifuged at 1,500 rpm for 5 min, resuspended in a solution of 90% FBS and 10% DMSO and maintained at − 80 °C until analysis. Samples were stained for 30 min at room temperature with the following primary antibodies for cell membrane markers conjugated with fluorochrome, at a 1:100 dilution in PBS: anti-CD117(c-Kit)-PE, anti-Sca1-FITC, anti-CD16/32-AF700, anti-CD34-eFluor660, anti-CD150-APC, anti-CD48-AF700 and Hematopoietic Lin Panel (CD11b, CD3e, CD45R/B220), Ly-6G, Ter-119)(all Life Technologies). After washing in PBS, when necessary samples were stained for 20 min with Pacific Blue-conjugated streptavidin (Life Technologies). Samples were then washed and resuspended in PBS for flow cytometry analysis. For HSC, LT-HSC, CMP, MEP and GMP analysis, gating was performed as described [53].

### Western blotting

Cells and tissues were collected and lysed in 40 μl RIPA buffer (20 mM Tris-HCl, pH 7.5, 150 mM NaCl, 1 mM EGTA, 1% NP-40, 1% sodium deoxycholate) supplemented with protease inhibitors (Roche) and phosphatase inhibitors (Sigma). Protein concentration was determined with the BCA Protein Assay (ThermoFisher). Protein extracts (30–40 μg) were mixed with Novex Sample Buffer and Reducing Agent (Life Technologies), resolved by SDS-PAGE in 12%, 10% or 4–12% gradient gels and blotted onto polyvinylidene difluoride membranes (Millipore). Membranes were blocked for 1 h with 5% non-fat dry milk (Bio-Rad) or 5% BSA in Tris-buffered saline containing 0.1% Tween 20 (TBS-T), and then incubated overnight at 4 °C with the following primary antibodies: mouse anti-β-actin (1:3000; Sigma-Aldrich); mouse anti-FABP4 (1:500, Santa Cruz Biotechnology); rabbit anti-adiponectin (1:1000; Thermo-Fisher Scientific); rabbit anti-Emilin-2 (1:1000 [45], antibody specificity shown in Additional file 1: Fig. S2F). After three washes for 10 min in TBS-T, membranes were incubated for 1 h with goat anti-mouse or anti-rabbit horseradish peroxidase-conjugated secondary antibodies (1:1000; Bethyl). After three further washes for 10 min in TBS-T, bands were detected with LiteAblot Extend chemiluminescent substrate (Euroclone), using a ImageQuantLAS 4000 digital imager (GE Healthcare).

### RNA extraction and RT-qPCR

RNA was extracted from cell samples using TRIzol reagent as recommended by the manufacturer (Invitrogen). cDNA was synthesized from 0.5 to 1 µm total RNA using M-MLV Reverse Transcriptase (Invitrogen). Real-time PCR reactions were performed using 5 × HOT FirePol EvaGreen qPCR Mix Plus (Solys) and run with RotorGene Q (Qiagen). Each sample was loaded at least in duplicate and analyzed with the RotorGene Q 2.0.24 software (Qiagen). Forward and reverse primers used for amplification are listed in Additional file 1: Table S1. *Rps16 *was used as internal control, in order to normalize differences in sample loading.

### Statistical analysis

Data are presented as mean ± s.e.m. The statistical significance was determined by unpaired two-tailed Student’s t test, and *P* values < 0.05 were considered as significant. One-way Anova with Student Newman–Keuls Post hoc test was used for the comparison of multiple groups.

## Supplementary Information

**Additional file 1: Figure S1.** Supporting immunofluorescence for Emilin-2 distribution, and specificity of the antibody against mouse murine Emilin-2. Related to Figs. 1, 2 and 3. (**A**) Digital reconstruction of sequential confocal microscopy images of Emilin-2 immunolabeling in femur sections of 6-month old wild-type mice. The red line on the left indicates compact bone area. Scale bar, 100 μm. (**B**) Magnification of vessels in BM of femur of 6-month old wild-type mice, following immunofluorescence for Emilin-2 (red) and Collagen IV (gray). Scale bar, 100 μm. (**E**) Immunodetection of Emilin-2 (green) in BM section of femur of wild-type and *Emilin2*^*−*/*−*^ mice. Nuclei were stained with Hoechst (blue). Scale bar, 50 μm. (**F**) Western blot for Emilin-2 in protein extracts of primary BM-MSC (left panel) and BM tissue (right panel) collected from wild-type and *Emilin2*^*−*/*−*^ mice. β-actin and red ponceau were used as loading controls for BM-MSC and BM tissue, respectively. WT, wild-type. **Figure S2.** Schematic diagram of the different experimental approaches used for culture, differentiation and analysis of MSC. Related to Figs. 2 and 3. (**A**) Summary of the experiments with ST2 cells shown in Fig. 2A–D. (**B**) Summary of the experiments with ST2 cells shown in Fig. 2E, F. (**C**) Comparison of Emilin-2 mRNA levels between the two initial conditions of approach A (UT t0) and approach B (day 3, UT t0), as determined by RT-qPCR in untreated ST2 cells. mRNA levels are shown as fold change compared to UT t0 condition (*n* = 3; **, *P* < 0.01). (**D**) Summary of the experiments with ST2 cells shown in Fig. 2F. (**E**) Summary of the experiments with primary murine BM-MSC cultures shown in Fig. 3. Adipogenic stimulus is indicated with a flash above the horizontal line. Sample analysis is indicated with a small histogram below the horizontal line. d, days. **Figure S3.** Supporting data for Emilin-2 deposition during adipogenic differentiation. Related to Fig. 3. (**A**) Immunodetection of Emilin-2 at different timing of adipogenic differentiation of WT BM-MSC. Nuclei were stained with Hoechst (blue). Scale bar, 50 μm. (**B**) Western blot analysis of Emilin-2, following SDS-PAGE in 4–20% gel, in primary BM-MSC at different time point during adipogenic differentiation. (**C**) Oil red O staining for lipid droplet after 18 days in undifferentiating condition (UT t18) or adipogenic differentiation (Adipo t18). Scale bar, 50 μm. **Figure S4.** Supporting data for flow cytometry analyses of BM from WT and *Emilin2*^*−*/*−*^ mice. Related to Figs. 4 and 5. Schematic diagram of flow cytometry lineage markers used for HSC and HPC identification. **Table S1.** Primers used for RT-qPCR analyses. Related to Experimental Procedures and Figs. 2 and 3.

## Data Availability

All data generated or analyzed during this study are included in this published article and its Additional file 1.

## Abbreviations

AML: Acute myeloid leukemia
BM: Bone marrow
BSA: Bovine serum albumin
CMP: Common myeloid progenitors
ECM: Extracellular matrix
FBS: Fetal bovine serum
GMP: Granulocyte myeloid progenitors
HPC: Hematopoietic progenitor cells
HSC: Hematopoietic stem cells
Lin^−^: Lineage-negative
LT-HSC: Long-term hematopoietic stem cells
MEP: Megakaryocyte–erythrocyte progenitors
MDS: Myelodysplastic syndromes
MSC: Mesenchymal stem cells
PBS: Phosphate-buffered saline
TBS-T: Tris-buffered saline containing 0.1% Tween 20
