# Supplementary material for: Emilin-2 is a component of bone marrow extracellular matrix regulating mesenchymal stem cell differentiation and hematopoietic progenitors
Source: Stem Cell Res Ther. 2022 Jan 10;13:2. doi: 10.1186/s13287-021-02674-2 (PMC8744352; doi:10.1186/s13287-021-02674-2)
Supplement: Supplementary file 1 — Additional file 1: Figure S1. Supporting immunofluorescence for Emilin-2 distribution, and specificity of the antibody against mouse murine Emilin-2. Related to Figs. 1, 2 and 3. (A) Digital reconstruction of sequential confocal microscopy images of Emilin-2 immunolabeling in femur sections of 6-month old wild-type mice. The red line on the left indicates compact bone area. Scale bar, 100 μm. (B) Magnification of vessels in BM of femur of 6-month old wild-type mice, following immunofluorescence for Emilin-2 (red) and Collagen IV (gray). Scale bar, 100 μm. (E) Immunodetection of Emilin-2 (green) in BM section of femur of wild-type and Emilin2−/− mice. Nuclei were stained with Hoechst (blue). Scale bar, 50 μm. (F) Western blot for Emilin-2 in protein extracts of primary BM-MSC (left panel) and BM tissue (right panel) collected from wild-type and Emilin2−/− mice. β-actin and red ponceau were used as loading controls for BM-MSC and BM tissue, respectively. WT, wild-type. Figure S2. Schematic diagram of the different experimental approaches used for culture, differentiation and analysis of MSC. Related to Figs. 2 and 3. (A) Summary of the experiments with ST2 cells shown in Fig. 2A–D. (B) Summary of the experiments with ST2 cells shown in Fig. 2E, F. (C) Comparison of Emilin-2 mRNA levels between the two initial conditions of approach A (UT t0) and approach B (day 3, UT t0), as determined by RT-qPCR in untreated ST2 cells. mRNA levels are shown as fold change compared to UT t0 condition (n = 3; **, P < 0.01). (D) Summary of the experiments with ST2 cells shown in Fig. 2F. (E) Summary of the experiments with primary murine BM-MSC cultures shown in Fig. 3. Adipogenic stimulus is indicated with a flash above the horizontal line. Sample analysis is indicated with a small histogram below the horizontal line. d, days. Figure S3. Supporting data for Emilin-2 deposition during adipogenic differentiation. Related to Fig. 3. (A) Immunodetection of Emilin-2 at different timing of a [file 13287_2021_2674_MOESM1_ESM.doc]

**Supplementary Figure S1.** **Supporting immunofluorescence for Emilin-2 distribution,** **and specificity of the antibody against mouse murine Emilin-2. Related to Figures 1, 2 and 3**. (**A**) Digital reconstruction of sequential confocal microscopy images of Emilin-2 immunolabeling in femur sections of 6-month old wild-type mice. The red line on the left indicates compact bone area. Scale bar, 100 μm. (**B**) Magnification of vessels in BM of femur of 6-month old wild-type mice, following immunofluorescence for Emilin-2 (red) and Collagen IV (gray). Scale bar, 100 μm. (**E**) Immunodetection of Emilin-2 (green) in BM section of femur of wild-type and *Emilin2*^–/–^ mice. Nuclei were stained with Hoechst (blue). Scale bar, 50 μm. (**F**) Western blot for Emilin-2 in protein extracts of primary BM-MSC (left panel) and BM tissue (right panel) collected from wild-type and *Emilin2*^–/–^ mice. β-actin and red ponceau were used as loading controls for BM-MSC and BM tissue, respectively. WT, wild-type.

**
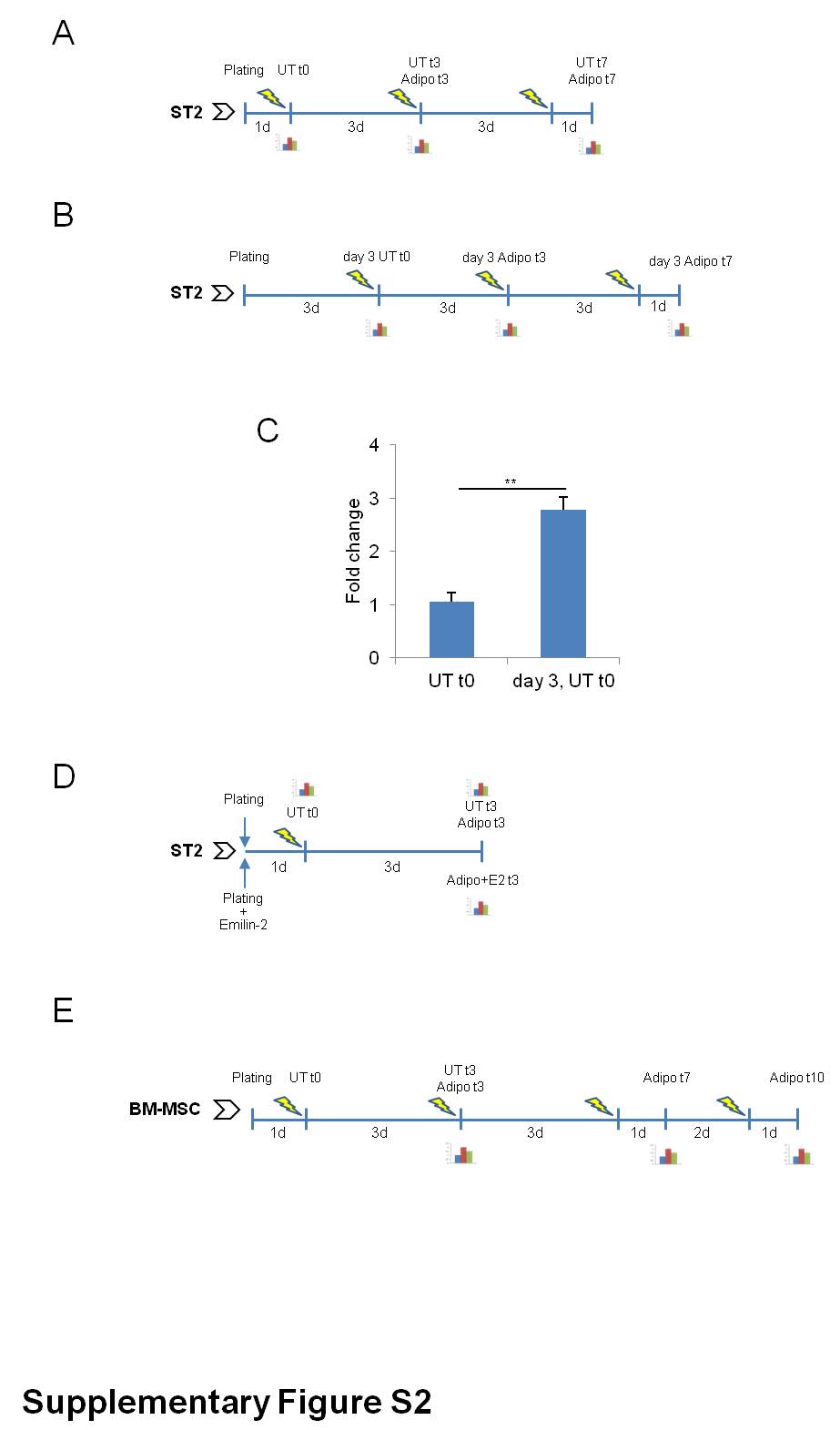
**

**Supplementary Figure S2. Schematic diagram of the different experimental approaches used for culture, differentiation and analysis of MSC. Related to Figures 2 and 3.** (**A**) Summary of the experiments with ST2 cells shown in Fig. 2A-D. (**B**) Summary of the experiments with ST2 cells shown in Fig. 2E, F. (**C**) Comparison of Emilin-2 mRNA levels between the two initial conditions of approach A (UT t0) and approach B (day 3, UT t0), as determined by RT-qPCR in untreated ST2 cells. mRNA levels are shown as fold change compared to UT t0 condition (*n* = 3; **, *P* < 0.01). (**D**) Summary of the experiments with ST2 cells shown in Fig. 2F. (**E**) Summary of the experiments with primary murine BM-MSC cultures shown in Fig. 3. Adipogenic stimulus is indicated with a flash above the horizontal line. Sample analysis is indicated with a small histogram below the horizontal line. d, days.

**Supplementary Figure S3. Supporting data for Emilin-2 deposition during adipogenic differentiation. Related to Figure 3. (A)** Immunodetection of Emilin-2 at different timing of adipogenic differentiation of WT BM-MSC. Nuclei were stained with Hoechst (blue). Scale bar, 50 μm. **(B)** Western blot analysis of Emilin-2, following SDS-PAGE in 4-20% gel, in primary BM-MSC at different time point during adipogenic differentiation. **(C)** Oil red O staining for lipid droplet after 18 days in undifferentiating condition (UT t18) or adipogenic differentiation (Adipo t18). Scale bar, 50 μm.

**
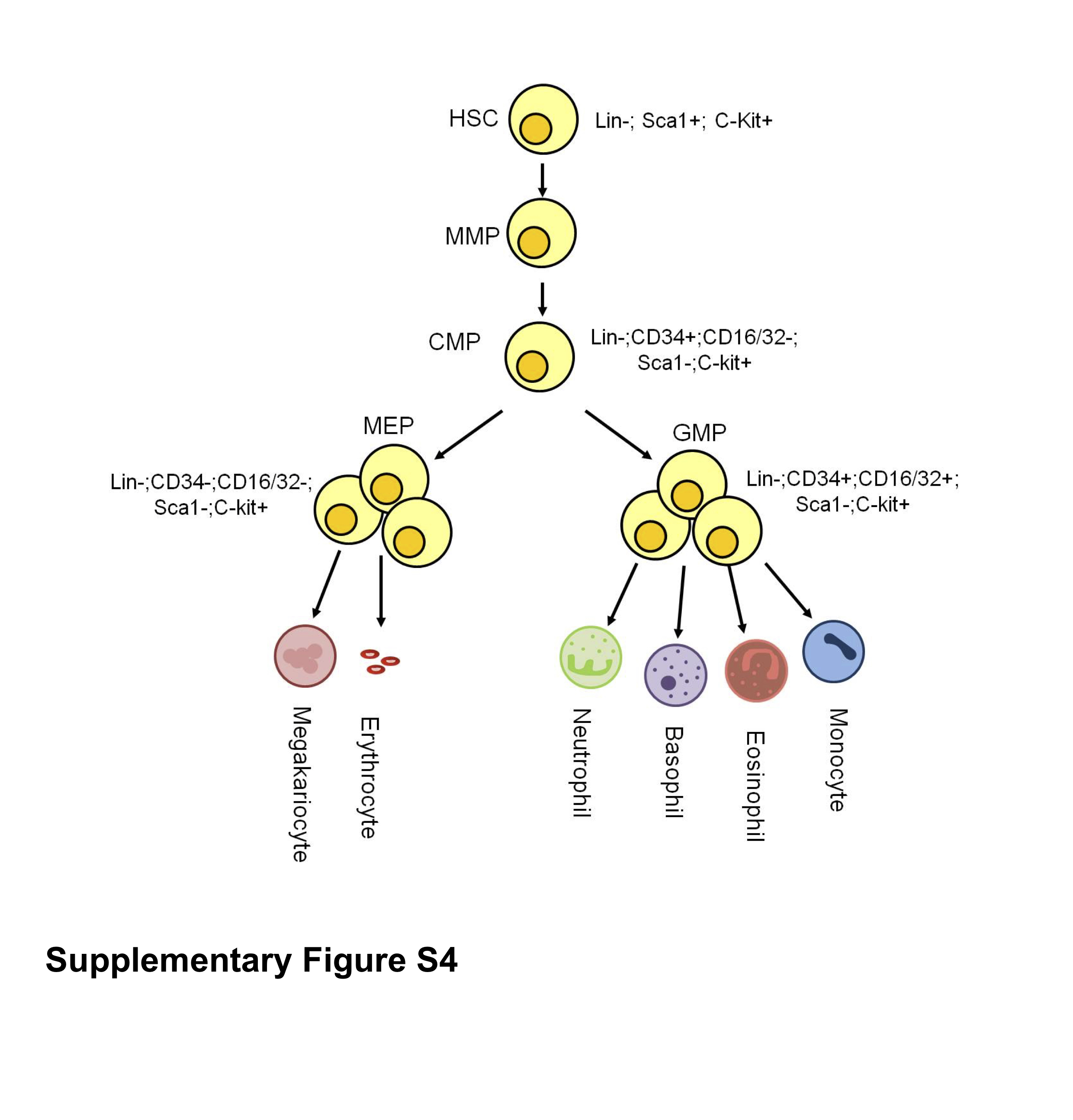
**

**Supplementary Figure S4. Supporting data for flow cytometry analyses of BM from WT and *Emilin2*^–/–^ mice.** **Related to Figure 4 and 5.** Schematic diagram of flow cytometry lineage markers used for HSC and HPC identification.

**Supplementary Table S1. Primers used for RT-qPCR analyses. Related to Experimental Procedures and Figures 2 and 3.**

| **Gene** | **Forward** (5’ -> 3’) | **Reverse** (5’ -> 3’) |
| --- | --- | --- |
| *Adipoq* | GGCATCCCAGGACATCCTG | GGACCAAGAAGACCTGCATC |
| *Cebpa* | TGCGCACCCCGACCTCC | TCTTGCGCACCGCGATGTT |
| *Emilin2* | CCCTGGTGTATCGGGTAAAC | ATGTGGTCTTTGGGACCTTCT |
| *Fabp4* | AGCACCATAACCTTAGATGGGG | CGTGGAAGTGACGCGTTTCA |
| *Rps16* | GCAGTACAAGTTACTGGAGCC | CGGTAGGATTTCTGGTATCG |
